# Molecular, genetic, and genomic basis of seed size and yield characteristics in soybean

**DOI:** 10.3389/fpls.2023.1195210

**Published:** 2023-11-15

**Authors:** Rupesh Tayade, Muhammad Imran, Amit Ghimire, Waleed Khan, Rizwana Begum Syed Nabi, Yoonha Kim

**Affiliations:** ^1^Upland Field Machinery Research Center, Kyungpook National University, Daegu, Republic of Korea; ^2^Division of Biosafety, National Institute of Agriculture Science, Rural Development Administration, Jeonju, Jeollabul-do, Republic of Korea; ^3^Department of Applied Biosciences, Kyungpook National University, Daegu, Republic of Korea; ^4^Department of Integrative Biology, Kyungpook National University, Daegu, Republic of Korea; ^5^Department of Southern Area Crop Science, National Institute of Crop Science, Rural Development Administration, Miryang, Republic of Korea

**Keywords:** seed size, seed weight, yield, quantitative trait, soybean

## Abstract

Soybean (*Glycine max* L. Merr.) is a crucial oilseed cash crop grown worldwide and consumed as oil, protein, and food by humans and feed by animals. Comparatively, soybean seed yield is lower than cereal crops, such as maize, rice, and wheat, and the demand for soybean production does not keep up with the increasing consumption level. Therefore, increasing soybean yield per unit area is the most crucial breeding objective and is challenging for the scientific community. Moreover, yield and associated traits are extensively researched in cereal crops, but little is known about soybeans’ genetics, genomics, and molecular regulation of yield traits. Soybean seed yield is a complex quantitative trait governed by multiple genes. Understanding the genetic and molecular processes governing closely related attributes to seed yield is crucial to increasing soybean yield. Advances in sequencing technologies have made it possible to conduct functional genomic research to understand yield traits’ genetic and molecular underpinnings. Here, we provide an overview of recent progress in the genetic regulation of seed size in soybean, molecular, genetics, and genomic bases of yield, and related key seed yield traits. In addition, phytohormones, such as auxin, gibberellins, cytokinins, and abscisic acid, regulate seed size and yield. Hence, we also highlight the implications of these factors, challenges in soybean yield, and seed trait improvement. The information reviewed in this study will help expand the knowledge base and may provide the way forward for developing high-yielding soybean cultivars for future food demands.

## 1 Introduction

**Figure 1:**
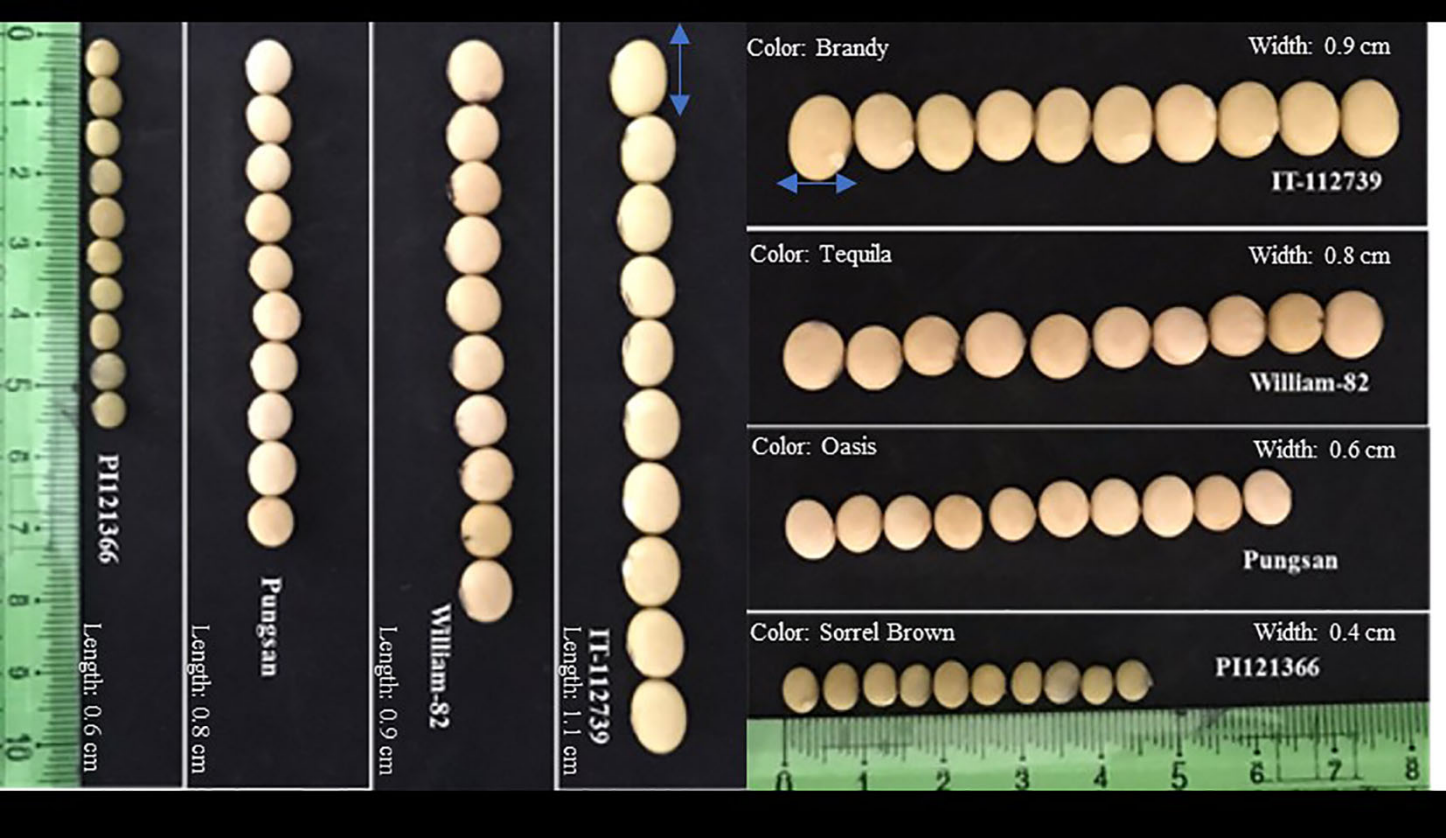
Different seed size soybean phenotypes (IT-112739, William-82, Pungsan, and PI1121366).

Many civilizations around the world consider seeds to be a staple diet. Seed grains, such as wheat (*Triticum aestivum*), rice (*Oryza sativa*), maize (*Zea mays*), and soybean (*Glycine max* L. Merr.), are regularly consumed by humans as a source of energy and nutrients. Global food security is continuously worsened by numerous drivers, such as climate change, declining cropland, rising human population, and, more recently, the COVID-19 pandemic and the Russia-Ukraine war (Ben Hassen and El Bilali, 2022; Lin et al., 2023). Furthermore, the yield of most staple food crops, including maize, wheat, rice, and soybean, is stagnating globally and may further decline with the increasing global temperatures (Zhao et al., 2017). Thus, increasing crop yield is a big challenge for the coming decades. Seed development is a complex process and involves diverse events, such as cell division, differentiation, and developmental regulatory processes. The seed developmental process influences the final seed size (SS) and morphology. Typically, SS is determined based on the seed’s thickness, length, and width and their ratios (**Figure 1**) (Cui et al., 2020). Seed width, height, and length impact the morphological integrity and quality of the seed. Given the importance of SS, many plant species established and demonstrated the molecular and genetic mechanisms involved in regulating SS and shape. Mainly, studies on model plant species like *Arabidopsis* (*Arabidopsis thaliana*) and barrel medic indicate that the molecular and genetic basis of SS depends on the early stages of seed development and involves different cellular events. These cellular events are initiated by zygotic division followed by the formation of nutritive tissues and the maturation stage, which subsequently accumulates storage products (carbohydrates, oil/lipids, proteins, vitamins, minerals, and nutrients) (Sreenivasulu and Wobus, 2013; Han et al., 2016).

Soybean is a top-rank economically important oilseed crop cultivated and consumed worldwide. Soybean seeds are excellent sources of energy, oil, protein, carbohydrates, and fat (Hudson, 2022). They are a rich, inexpensive source of protein for humans and an essential source of fodder/feed for domesticated livestock. Consequently, soybean is regarded as one of the most commercially significant oilseed crops. The cultivated soybean has a long history of domestication and breeding from wild soybeans (*Glycine soja* Sieb. and Zucc.) (Hymowitz, 1970; Caldwell and Howell, 1973). Domestication and selection have undoubtedly resulted in modern soybean cultivars with superior seed characteristics, such as seed color, size, shape, and weight, compared to their wild progenitors (Liu et al., 2020d; Bohra et al., 2022). Most seed domestication and improvement traits are directly related to crop yield. In addition, seed-based products, such as soya tofu, sprout, edamame, and natto, vary with SS characteristics. Thus, SS and seed weight (SW) are essential traits in soybean breeding for crop yield improvement. The yield is a complex trait governed by multiple genes/loci or minor quantitative trait loci (QTLs) and influenced by environmental factors. The genetic makeup and molecular mechanisms underlying the seed traits are crucial for identifying gene regulatory networks of SS and yield. In cereals like rice and maize, genetic and molecular controls of seed development and yield are well established. The research on cereal seed traits leads to significant yield enhancement compared to soybean. However, basic research on the seed and embryo development of legumes, including soybean, medicago (*Medicago truncatula*), common bean (*Phaseolus vulgaris*), pea (*Pisum sativum*), and broad bean (*Vicia faba*) was reported (Le et al., 2007).

Recent research has employed genomic technologies, including genomics, proteomics, and transcriptomics, to uncover QTLs, genes, and specific underlying pathways that are critical for leguminous seed development (Gao et al., 2018; Afzal et al., 2020; Liu et al., 2022). More than 500 QTLs specific to soybean and associated with yield-related traits (SW, 100-seed weight (HSW), number of branches on main stem, plant height, total seed number per plant, number of nodes, total pod number per plant (NPP), seed yield per plant, and diameter of main stem) have been revealed on the Soybase Genome Database (SoyBase, 2023). In addition, previous studies in soybean reported crucial genes (*GmFAD3*, *GA20OX*, *GmLEC2*, *GmPDAT*, *GmKIX8-1*, *GmSSS1*, *ST1*, and *GmGA3ox1*) that alter SS and yield by regulating lipid accumulation, cell expansion, and cell proliferation (Manan et al., 2017; Liu et al., 2020a; Nguyen et al., 2021; Hu et al., 2022; Li et al., 2022b; Zhu et al., 2022). Furthermore, researchers identified and reported genetic factors that influence SS and SW in soybean (Zhou et al., 2016; Jing et al., 2018). Similarly, (Gao et al., 2018) discovered several genes involved in soybean seed development, accumulation of storage proteins and oils, and seed coat development. However, although genetic and genomic resources are available for soybean, information on yield and underlying genes for most reported QTLs has not been extracted. Thus, broadening our knowledge of genetics and genomics can provide insight into the molecular, genetic, and genomic bases of seed yield in soybean and identify genes and pathways essential for seed development. This knowledge can then be used to breed soybean cultivars with improved seed yields through traditional or advanced molecular breeding approaches.

Overall, molecular, genetic, and genomic studies of soybean seed development as well as related traits are essential for understanding the underlying biological processes and developing strategies to increase seed yield in this important crop. Although the genetics and genomics of seed development and yield in soybean have been the subject of many studies in recent years, there is no complete compiled information on the genetic and genomic basis of the key seed yield traits. Thus, this review aims to provide an overview of recent progress in molecular, genetic, and genomic levels in the context of yield and related key seed yield traits. In addition, several phytohormones, such as auxin, gibberellins (GA), cytokinins (CK), and abscisic acid (ABA), regulate SS and yield in soybeans; therefore, we also highlight the implications of these factors in our review. Finally, challenges in soybean yield and seed trait improvement and avenues to expand the knowledge base and its significance in soybean crop genetic improvement are addressed.

## 2 Key seed yield traits and significance for crop improvement

Seed yield is an essential trait in crops because it determines the number of seeds a plant produces, which can be used for food, feed, or propagation. Improving seed yield can help increase crop production and enhance food security. Several factors can affect seed yield in crops, including genetics, environment, and management practices.

Soybean seed yield is also a complex quantitative trait governed by multiple genes, broadly influenced by the growing conditions and latitudes. Although soybean is cultivated globally, it is a short-day, photoperiod-sensitive crop with different geographical latitude ranges (Chen et al., 2020a; Toleikiene et al., 2021). Soybean seeds vary in size, shape, length, and color, as displayed in **Figure 1**. Seed yield is a complex trait determined by various components. Similarly, a complex features that are influenced by both genetic and environmental factors during seed growth and maturation include seed size, oil content, and protein content. Given their significance in soybean breeding, researchers have used numerous bi-parental derived populations, including the F2 population, recombinant inbred lines (RILs), chromosome segment substitution lines (CSSLs), and near-isogenic lines (NILs), to perform extensive linkage analysis to QTL associated with these three seed traits (Qi et al., 2014; Warrington et al., 2015; Yang et al., 2019; Yang et al., 2022). In the SoyBase Genome Database, hundreds of QTLs associated with SS (including SW), oil accumulation, and protein content have so far been identified. For instance, 396 QTLs are associated with SW and SS, 333 with seed oil content, and 234 with seed protein content. Some of these QTLs, including those relating to protein content, oil accumulation, and seed size, had overlapping areas, indicating the presence of pleiotropic regulating genes in these QTLs (Wang et al., 2015a; Cui et al., 2020; Kumawat and Xu, 2021; Kumar et al., 2022; Luo et al., 2022). Additionally, characteristics like SS, SW, NPP, number of seeds per pod, and HSW are key component traits directly influencing crop yield. In some studies, SS is considered as SW and vice versa (Xu et al., 2022). The same study revealed how the size of seeds affects the yield. It was found that bigger seeds didn’t make the yield better; in fact, they sometimes made the yield lower compared to smaller seeds, especially in good growing conditions. Similarly, another study reported a negative correlation of HSW (*r*= -0.05) with yield while a significant positive correlation of pod number (*r*= 0.44), and total grain number (*r* = 0.44) was noticed with the yield output (Attipoe et al., 2023) while contrastingly, some studies have revealed different results. A study conducted to check the effect of SS on crop yield, including others, has revealed a positive impact, where larger seeds produced an increase of 5.4% on average from the small seeds genotypes in 10 experiments (Smith and Camper, 1975), furthermore, a separated study also revealed high crop yield for the accessions having larger SS comparatively to the smaller genotypes (Adebisi et al., 2013), adding more, another study conducted revealed NPP, total number of seeds, and HSW showed a positive correlation of (*r* = 0.92), (*r* = 0.93), and (*r* = 0.063) with yield, respectively (Vu et al., 2019). Analyzing the mentioned studies, it can be concluded that the most important and defining traits contributing to crop yield directly are the pods number and number of seeds, revealing the biggest correlation with the yield in every study conducted (Adebisi et al., 2013; Attipoe et al., 2023). However, relying solely on simple correlation to establish the causal relationship between yield components and yield might not accurately capture the intricate cause-and-effect dynamics, thus leading to potential inefficiencies in the selection strategy. Although the SS is one of the factors that influence the crop’s overall yield directly, it also has a wide indirect influence on the crop output such as larger seeds are noted to be heavier (Alonso-Blanco et al., 1999) which also means more stored energy reserves, larger cotyledons, and greater initial vigor, ultimately resulting in good overall crop production and yield output (Ambika et al., 2014). On the other hand, traits like plant height, number of nodes, branches, and growth also indirectly influence crop yield. Adding to that, research reported SS as a key yield component, where it stated; it influences seed thickness, length, and width (Alonso-Blanco et al., 1999). In addition, it was reported that contrary to stress-associated traits, the domestication and development of soybeans through both natural and artificial selection resulted in an increase in genetic variation for traits related to seed protein, oil content, flowering, SW, and total yield (Hwang et al., 2014; Lu et al., 2020; Miao et al., 2020). *Arabidopsis* and cereal crops are well studied and documented for yield traits compared to soybean. For example, in rice, several seed-size regulatory genes have been cloned and characterized (Li and Li, 2016; Duan and Li, 2021; Chen et al., 2022). However, the quantitative nature of the trait impedes the discovery and functional characterization of the underlying regions/loci and genes in soybean. In this regard, the most recent studies on the genetic basis, genomic regions/loci, QTLs, and genes associated with soybean yield attributes may help us better understand the molecular underpinnings determining soybean seed yield. This knowledge could lead to the best exploration of traits and associated genes through molecular breeding strategies for greater yield enhancement in soybean.

## 3 Molecular genetic and genomics basis of soybean yield traits

In the recent decade, dissecting the genetic basis of soybean yield traits has drawn growing interest. The development of advanced technologies such as Next Generation Sequencing (NGS); enables researchers to obtain comprehensive genomic information by rapibly sequencing large amounts of DNA. This has been instrumental in the development of high-resolution genetic linkage maps, allowing for detailed investigations into desired agronomic traits (Singh et al., 2015) and, Clustered Regularly Interspaced Short Palindromic Repeats (CRISPR) technology has provided a powerful tool for precise genome editing, enabling researchers to elucidate gene functions and dissect the genetic basis of complex traits. By utilizing CRISPR-Cas9, scientists have been able to investigate the role of candidate genes identified through transcriptome and proteome analyses, as well as study mutants and transgenic plants to gain insights into the mechanisms underlying soybean yield traits (Ran et al., 2013). CRISPR/Cas9 has been used in 20 crop species, improving traits like yield and resistance to biotic and abiotic stresses (Chaudhary et al., 2022). As mentioned earlier, yield is a quantitative trait governed by multiple genes and influenced by small-effect QTLs and environmental conditions. Thus, investigating the yield traits and identifying the undermining genetic and molecular basis of these traits is very challenging. However, ongoing advances in genomics and molecular biology and the availability of pangenome of wild and cultivated soybeans have facilitated the identification of major and minor QTLs, genomic regions, and candidate genes associated with yield traits in soybean (Tayade et al., 2019; Liu et al., 2020e; Li et al., 2022d). Researchers have developed high-resolution genetic linkage maps based on single nucleotide polymorphism (SNP) markers to study the desired agronomic traits (Song et al., 2016; Zhang et al., 2018; Knizia et al., 2021). By keeping in mind the complex nature of traits, several studies developed biparental mapping populations to dissect the genetic bases of yield and related traits, which led to the identification of several essential QTLs for regulatory genetic variation associated with seed yield in soybean (Li et al., 2020; Li et al., 2022c).

In comparison to the method of gene identification related to specific traits using biparental QTL mapping, a more precise location of QTLs can be obtained from genome-wide association studies (GWAS) (Zeng et al., 2017). Researchers used the GWAS approach to identify genomic regions associated with soybean yield traits. Additionally, candidate genes have been identified through transcriptome and proteome analyses and functional studies of mutants and transgenic plants. While it is not our objective to fully explain all the QTLs or candidate genes identified through transcriptome and proteome, we highlight some of the key characteristics and QTLs of yield traits in the following part.

### 3.1 Unraveling the complex genetic architecture of yield-related QTLs in soybean

**Figure 2:**
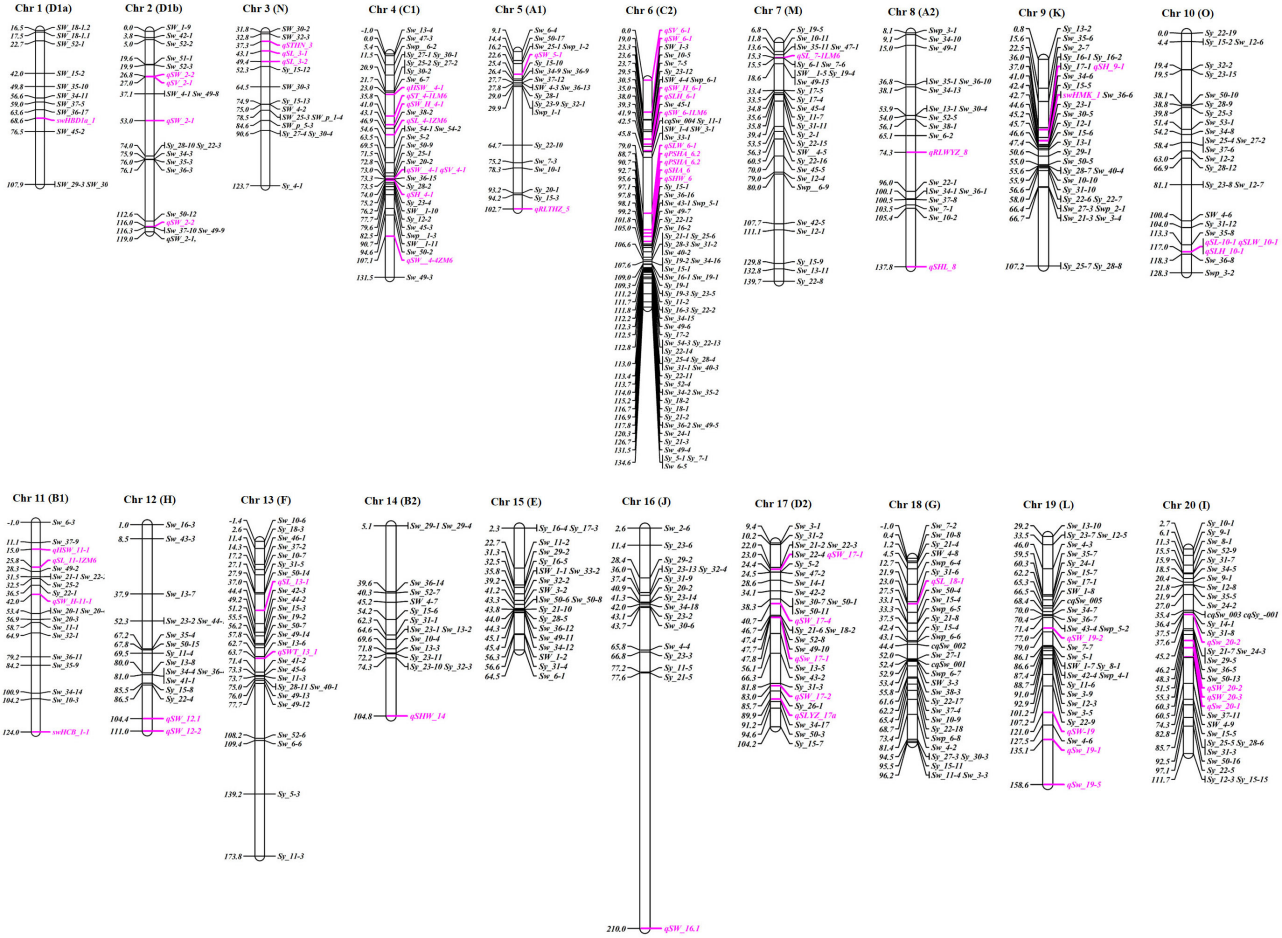
Chromosomal distribution of important QTLs associated with the seed yield traits throughout the soybean genome. QTLs reported on SoyBase are mapped on chromosomes. Individual QTLs explaining >10% of phenotypic variation for the respective yield traits are highlighted in pink. They include seed volume (*SV*), seed length (*SL, SLYZ*), seed width (*SW^a^*), seed height (*SH*), seed length-to-height ratio (*SLH*), seed length-to-width ratio (*SLW*), seed width-to-height ratio *(SWH*), seed hilum width (*SHW*), seed hilum length (SHL), seed hilum area (SHA/PSHA), seed thickness (*ST/STHN*), seed weight (*sw, SW^b^, SWT^b^, swHCB, swHMK*, and *swHBD*), and hundred-seed weight (*100SW*).

Several traits are considered essential and directly impact the overall yield of the crop. As previously discussed, SS, SW, shape, and HSW are important yield component traits. Although these are the main elements determining crop yield, most earlier investigations focused on main-effect QTLs for SS, morphology, and HSW in soybean. Globally, numerous studies have been conducted on the QTL localization of soybean seed yield traits, especially for SS and SW. Compared to studies on SW, studies on QTL localization for SS were fewer. So far, 304 QTLs have been reported for SW, whereas, <100 QTLs have been reported for SS (seed shape, length, width, and volume/thickness) on the Soybase. However, some researchers recently reported additional multi-environment QTLs using the different genetic backgrounds for SS and weight, which may not be listed in the soybean database. Mian et al. (1996) reported 16 QTLs for SS and shape on 12 soybean chromosomes in this context. However, they used low throughput outdated restriction fragment length polymorphisim (RFLP) markers. Similarly, 27 QTLs for SS and 3 QTLs for seed length were identified using the low-density simple sequence repeats (SSR) markers in different mapping populations of soybean (Hoeck et al., 2003; Li et al., 2008). Further, Lu et al (2017) identified 19 main-effect QTLs and three epistatic-effect QTLs for seed length. Sun et al (2022) identified 10 QTLs using the RIL population for SS in four environments with phenotypic variations (PVE) ranging from 3.6% to 9.4%. Similarly, 53 and 27 QTLs were identified for SS and seed shape, respectively, in three growing seasons using the RIL population created by crossing K099 (small SS) × Fendou 16 (large SS) parents. Among them, six QTLs (*qSW8.1, qSW16.1, qSLW2.1, qSLT2.1, qSWT1.2*, and *qSWT4.3*) displayed a significant range of logarithm of odd (LOD) of 3.80–14.0 and PVE of 2.36%–39.49% in at least two growing seasons (Kumawat and Xu, 2021). Luo et al. (2023) recently identified 60 QTLs for SS and 25 QTLs for SW using the RIL population in five natural environments and their combined environment. For the seed yield, 365 QTL loci have been reported in the SoyBase. QTLs for SS and SW are represented in **Figure 2** from recently published articles and the soybean database, and major QTLs for SS and SW with a PVE >10 are reviewed in **Table 1**. Another crucial yield-related attribute is HSW. During domestication, HSW was one of the fitness traits and selection parameters for soybean (Duan et al., 2022; Goettel et al., 2022). Previously, researchers found wide variation in the HSW in the American and Chinese germplasms ranging from 5.64 to 34.80 g (Zhang et al., 2016a; Zhao et al., 2019). To determine the genetic factor for the variation of HSW, linkage mapping was conducted with multiple backgrounds, and associated QTLs were identified. For example, 19 QTLs were identified for HSW in individual and combined environments, among which seven novel minor (*R*^2^ < 10%), 2 (*qSW-17-1* and *qSW-17-4)* major (*R*^2^ > 10%), and eight stable QTLs (Karikari et al., 2019). Recently, 3 major QTLs (*q100SW-4-1, q100SW-11-1*, and *q100SW-17-1*) with PVEs of 11.41%, 14.67%, and 14.37%, respectively, were identified (Kumar et al., 2023).

**Table 1:** Major quantitative trait loci (QTLs) discovered contributing to > 10% phenotypic variation for the yield traits in soybean.

Traits	Population	QTL	Chr (LG)	Marker Interval	MP (cM)	LOD	PVE (%)	Reference
Seed Size	F_2_ and F_2_:_3_ (‘AGS 457’ × ‘SKAF 148’)	*qSV-6-1*	6 (C2)	Sat_062-Sat_153	0.00	2.6	15.64	(Kumar et al., 2023)
		*qSV-2-1*	2 (D1b)	Satt558-Satt266	27.00	3.23	13.42	
		*qSV-4-1*	4 (C1)	AW277661-Sat_322	73.00	12.34	33.94	
		*qSL-10-1*	10 (O)	Satt592-Sat_341	117.00	4.71	13.70	
		*qSL-18-1*	18 (G)	Satt288-Sat_164	23.00	2.87	17.21	
		*qSL-13-1*	13 (F)	Sat_090-Satt656	37.00	3.18	22.84	
		*qSW^a^-6-1*	6 (C2)	Sat_062-Sat_153	19.00	3.12	17.56	
		*qSW^a^-2-1*	2 (D1b)	Satt266-Satt282	53.00	8.21	17.09	
		*qSW^a^-2-2*	2 (D1b)	Satt274-Satt459	116.00	3.78	12.85	
		*qSW^a^-4-1*	4 (C1)	AW277661-Sat_322	73.00	7.58	14.15	
		*qSLW-10-1*	10 (O)	Satt592-Sat_341	117.00	6.97	20.66	
		*qSLH-10-1*	10 (O)	Satt592-Sat_341	117.00	2.55	12.67	
		*qSLH-6-1*	6 (C2)	Sat_153-Satt322	38.00	3.32	18.23	
		*qSLH-10-1*	10 (O)	Satt592-Sat_341	117.00	4.92	16.67	
		*qSWH-4-1*	4 (C1)	Satt578-Satt136	41.00	3.21	12.21	
		*qSWH-6-1*	6 (C2)	Sat_153-Satt322	35.00	3.45	11.42	
		*qSWH-11-1*	11 (B1)	Sat_331-Satt359	42.00	4.12	18.27	
		*qSH-4-1*	4 (C1)	AW277661-Sat_322	74.00	5.2	17.52	
		*qSH-9-1*	9 (K)	Satt178-Satt337	37.00	3.13	17.28	
		*qSLW-6-1*	6 (C2)	Satt322-Sat_246	79.00	3.31	10.95	
	RIL (‘Guizao1’× ‘B13 (GB13)’)	*qSL-3-1*	3 (N)	bin32-bin43	43.10	11.45	15.91	(Luo et al., 2023)
		*qSL-3-2*	3 (N)	bin51-bin55	49.40	10.38	14.56	
		*qSW^a^-20-2*	20 (I)	bin46-bin53	51.50	8.29	11.80	
		*qSW^a^-20-3*	20 (I)	bin64-bin68	55.30	15.04	21.93	
	RIL (‘BD2’×”BX10’)	*qSLYZ-17a*	17 (D2)	*Chr17.38929104*	89.93	4.33	11.20	(Cui et al., 2020)
		*qRLTHZ-5*	5 (A1)	Chr05.41789089	102.73	5.51	14.00	
		*qRLWYZ-8*	8 (A2)	Chr08.13315448	74.34	5.16	13.20	
		*qSTHN-3*	3 (N)	*Chr03.38579634-Chr03.38860016*	*37.27*	*5.61*	*26.20*	
	F_2:3_ (‘Pak Chong 2’ × ‘Laos 7122’)	*qSW^a^-6-1_LM6_*	6 (C2)	bin597	41.91	4.15	10.70	(Moongkanna et al., 2011)
	RIL (‘Kefeng1’ × ‘Nannong1138-2’)	*qSL-7-1_LM6_*	7 (M)	bin744-bin745	15.25	4.901	13.70	(Hu et al., 2013)
	RIL (‘K099 (small seed size)’ × ‘Fendou 16 (large seed size)’)	*qSW^a^16.1*	16 (J)	Chr16_36298773-Chr16_36530800	210	5.8-14.00	14.74-23.00	(Kumawat and Xu, 2021)
	RIL (‘JD12’ × ‘NF58’)	*qSHW6*	6 (C2)	Gm06_24186496-41558653	95.62	6.43	15.60	(Zhao et al., 2021)
		*qSHW14*	14 (B2)	Gm14_46973150-49265151	104.77	4.27	10.60	
		*qSHL8*	8 (A2)	Gm08_44057851-44602360	137.77	4.75	11.80	
		*qSHA6*	6 (C2)	Gm06_17617727-24186496	92.66	5.97	14.50	
		*qPSHA6.2*	6 (C2)	Gm06_17194761	88.70	4.79	11.80	
		*qPSHA6.2*	6 (C2)	Gm06_17194761-17617727	90.70	5.89	14.40	
Seed weight	RIL (‘Nannong493-1 (*G. max*)’ × ‘PI 342618B (*G. soja*)’)	*qSW^b^-17-1*	17 (D2)	bin3613-bin3621	23.01	7.28	13.20	(Karikari et al., 2019)
		*qSW^b^-17-4*	17 (D2)	bin3645-bin3651	40.71	5.96	11.58	
	RIL (‘PI595843 (PI)’ × ‘WH’)	*qSw-19-1*	19 (L)	bin4518-bin4523	135.11	10.89	11.60	(Xu et al., 2023)
		*qSw-19-5*	19 (L)	bin4552-bin4555	158.61	11.36	11.42	
		*qSw -20-2*	20 (I)	bin4647 -bin4649	37.61	13.48	13.33	
	RIL (‘Zhongdou27’ × ‘Jiunong20’)	*qSW^b^2-1*	2 (D1b)	Sat_198-Satt274	118.98	4.21	14.56	(Wu et al., 2018)
		*qSW^b^2-2*	2 (D1b)	Satt157-sat_135	26.80	4.23	12.54	
		*qSW^b^5-1*	5 (A1)	Satt276-sat_265	22.60	4.38	13.99	
	RIL (‘Hefeng25’ × ‘Conrad’)	*swHCB1-1*	11 (B1)	satt453–sat123	123.95	7.65	31.09	(Han et al., 2012)
	RIL (‘Hefeng25’ × ‘Maple Arrow’)	*swHMK-1*	9 (K)	satt555–satt046	42.70	3.06	23.89	
	RIL (‘Hefeng25’ × ‘Bayfield’)	*swHBD1a-1*	1 (D1a)	Satt198–Satt168	68.62	5.74	32.76	
	RIL (‘PI 483463’ × ‘Hutcheson’)	*qSW^b^17-2*	17 (D2)	BARC-025927-05161-BARC-013709-01242	83.00	6.38	12.23	(Kulkarni et al., 2016)
		*qSW^b^19-2*	19 (L)	BARC-016145-02292-BARC-065769-19741	77.00	5.70	10.39	
	RIL (‘Zhongpin03-5373 Zhonghuang13’)	*qSW^b^-12-2*	12 (H)	satt637-satt142	111.00	9.44	10.51	(Liu et al., 2013)
		*qSW^b^-19*	19 (L)	Map-3948-Map-3960	121.00	10.67	10.98	
	F_2_ (‘Jidou 12’ (G. max) × ‘ZYD2738’ (G. soja)), F*_2_:_3_(‘Jidou 9’ (G. max) × ‘ZYD2738’ (G.soja)*	*qSWT^b^_13_1*	13 (F)	Satt114	63.68	5.51-9.6	21.2-39.5	(Yan et al., 2014)
	OA-RILs (‘Ohsuzu’ × ‘Athow (PI 595926)’ and ST-RILs(‘Stressland (PI 593654)’ × ‘Tachinagaha (PI 561396)’)	*qSw17-1*	17(D2)	GMES4177	47.80	3.6-11.8	11.4-15.0	(Kato et al., 2014)
		*qSw20-1*	20 (I)	Sat_105	60.30	18.5	20.3-27.0	
	BC_3_F_5_ (‘Jackson’ × ‘JWS156-1’)	*qSW^b^12.1*	12 (H)	Sat_180	104.37	6.78-12.31	15-25-23.25	(Liu et al., 2018)
	F_2_ and F_2_:_3_ (‘AGS 457’ × ‘SKAF 148’)	*q100SW-4-1*	4 (C1)	Sct_186-Satt578	23.00	3.82	11.41	(Kumar et al., 2023)
		*q100SW-11-1*	11 (B1)	Satt484-Satt453	15.00	3.49	14.67	

QTL, Quantitative trait loci; Mp, Mapped position; LOD, logarithm of the odds; PVE, percentage of phenotypic variation.

SW ^a^ = Seed width.

SW ^b^= Seed weight.

Seed Size: volume, length, width, height, length-to-height ratio, length-to-width ratio, width-to-height ratio, hilum width, hilum length, hilum area, and thickness.

Seed weight: seed weight and hundred-seed weight.

Regarding the NPP, several QTLs have been identified using diverse genetic backgrounds and environments, indicating that the variation in pod number is widespread in soybean. Researchers demonstrated that increased pod setting per plant and NPP highly influence the soybean yield; however, it is sensitive to the environment controlled by multiple genes, most of them with small effects (Xavier and Rainey, 2020). In order to improve the effectiveness of breeding for higher yields while taking the significance of the total NPP into mind, several studies identified QTLs linked to the total number of pods (Zhang et al., 2010; Rodrigues et al., 2016; Liu et al., 2017b). To date, 51 QTLs were reported in the SoyBase for the NPP. **Figure 2** displays the identified QTLs, excluding QTLs whose position was not determined. Recently, Sun et al. (2022) identified the 6 and 5 QTLs for total flower pod numbers (TFPN) and NPP, respectively. Furthermore, among the 11 QTLs, they identified QTLs (*qFPN4)* with PVEs of 9.2% and 9.6% for TFPN and NPP, respectively, on chromosome 4.

Previous research primarily concentrated on discovering significant QTLs for SS/SW under various genetic backgrounds. However, soybean linkage mapping, particularly for yield attributes, still needs to be researched, as several studies have been unable to discover stable QTLs and lack a thorough examination of epistasis and environmental impacts.

### 3.2 Quantitative trait nucleotides for seed yield traits unveiled by GWAS; exploring genetic association

GWAS have become a common approach to finding out marker-trait associations (MTAs) for complex traits in the genetically diverse population of plants and detecting the relationship between the genetic variance and these traits (Visscher et al., 2017; Kaler et al., 2020). In addition to perform QTL mapping to analyze soybean yield traits such as SS/SW and HSW, GWAS was undertaken to discover QTLs, quantitative trait nucleotides (QTNs), and the genetic loci associated with these traits. In association analysis, mostly natural populations or germplasm resources are taken as the research object, based on linkage disequilibrium, and millions of SNP markers apply for correlation analysis of genetic factors of desired traits. Compared with linkage analysis, association analysis is fast, high-throughput, and displays a high-resolution advantage. With the recent advancements in powerful statistical genetics models, the adjustment of false discovery rates, and the use of improved population structure, kinship matrices, and computational tools, GWAS became an efficient method for QTN detection for desired traits. GWAS, QTLs related to 140 different soybean traits, such as root, shoot, yield, nutrient constituents, and biotic and abiotic stresses, are listed in the SoyBase (https://www.soybase.org/GWAS/list.php). Thus, it is clear that GWAS QTL research in soybean is increasing.

In soybean, this approach is used for MTAs and the identification of QTLs or QTNs For example, Li et al (2019b) identified 139 QTNs linked with four yield-related traits using 82,187 SNPs by phenotyping 133 soybean landraces. Among them, 35 QTNs were repeated across evaluated environments. Similarly, Qi et al. (2020) identified 118 QTNs associated with the HWS of soybean, using 109,676 SNP markers from 144 RILs. Ayalew et al. (2022) used the largest number of diverse germplasm resources (541) and 50K SNPs, which led to identifying 19 QTNs significantly associated with seed yield. Their study found two stable seed yield QTNs on chromosomes 9 and 17 detected consistently in three environments. In addition, using 6K SNP markers and 470 soybean accessions, 14 QTNs were identified for seed yield-related traits on 6 different chromosomes (Jo et al., 2022). Similar studies with significant effects on seed yield traits in a selected population of soybean germplasm were reported (Zhang et al., 2016a; Yan et al., 2017; Copley et al., 2018; Li et al., 2019a; Li et al., 2019c). In **Table 2**, we have provided the major GWAS QTNs reported in the last decade, displaying significant LOD and contributing to higher PVE for yield traits, all tested in two or more environments.

**Table 2:** Reported genome-wide quantitative trait nucleotides (QTNs) for major seed yield traits in soybean.

SNP ID/Marker	Chr (LG)	Position (bp)	Traits	R^2^ (%)	Reference
Gm6_Hap29a	6 (C2)	15115808	SY	21.00	(Contreras-Soto et al., 2017)
Gm12_Hap42a	12 (H)	5610868		12.10	
Gm03-43266860	3 (N)	43266860	SW	10.37	(Zhang et al., 2015; Contreras-Soto et al., 2017; Yan et al., 2017; Hu et al., 2020)
AX-93713187	4 (C1)	49453295		7.51-8.27	
AX-93713188	4 (C1)	49453902		7.51-8.27	
Gm04_37010886	4 (C1)	40151473		9.0-12.80	
Gm04_27912357	4 (C1)	36376946		9.6-11.1	
Gm5_Hapl Oa	5 (A1)	9012813		13.80	
Gm7_Hapl 3a	7 (M)	6604493		14.80	
Gml1_Hap13a	11 (B1)	5065170		13.20	
Gm12_Hap42b	12 (H)	5610878		31.20	
Gm12_Hap42b	12 (H)	5610878		21.80	
Gm13-33,852,022	13 (F)	33852022		10.88	
Gm17_2500333	17 (D2)	2492594		7.9-10.6	
Gm17_8635426	17 (D2)	8364226		7.5-13.20	
AX-116905453	2 (D1b)	44205396	HSW	8.42-15.07	(Qi et al., 2020)
AX-157126994	6 (C2)	49959758		6.35-14.03	
AX-157388275	8 (A2)	46069989		7.74-11.96	
Chr04_13518076	4 (C1)	13518076	SS	–	(Fang et al., 2017)
Chr04_13890053	4 (C1)	13890053			
Chr04_14176459	4 (C1)	14176459			
Chr13_21924051	13 (F)	21924051			
ss244709037	2 (D1b)	ss244709037	HSW, SL, and SWD	10.18-10.31	(Li et al., 2019a)
ss246792949	9 (K)	3697352		10.05-10.93	
ss248666643	15 (E)	17219745		9.16-13.87	
BARC-024093-04723	11 (B1)	38631243	TSPP and PPP	7.89, 9.13	(Zhang et al., 2015)
Map-0676	4 (C1)	42913914	HPFW	8.55-11.42	(Li et al., 2021)
Q-07-0082152	7 (M)	8283800		7.71-11.05	

SY, seed yield; SW, seed weight; HSW, 100-seed weight; SS, seed size, HSW, SL, seed length; SWD, seed width; TSPP, total seed per plant; PPP, pod per plant; HPFW, 100 pods fresh weight.

### 3.3 Key genes governing yield-related traits in soybean

Genes governing particular traits are always a topic of interest for plant breeding programs since most crop yield-related traits are governed by numerous genes (Zhang et al., 2020). Although QTL mapping and GWAS for yield traits are conducted in soybean, it remains challenging to effectively boost yield or quality by genetic modification due to the lack of stable QTLs and functional genes. Discovering QTLs and genes is the first step toward understanding the molecular basis of yield and a necessary step for establishing efficient marker-assisted breeding technology and conducting gene identification and editing.

With the recent advancement in genomic tools and DNA sequencing, crop production, yield-related traits, and their respective genomic location, can be identified more precisely and faster (Ravelombola et al., 2021). Several genes governing the soybean’s productivity or seed yield have been identified to date. For example, the *BIG SEEDS1 (BS1)* gene involved in controlling SS and SW has been reported and described in *Medicago truncatula* and soybean (Ge et al., 2016), where it is also involved in monitoring the size of pods and leaves. Similarly, *GmCYP78A10b* regulates the SS, width, and thickness in soybean with 7.2% of the variation in the SW (Wang et al., 2015b). *GmCYP78A72* is involved in the increase in SS (Zhao et al., 2016), *GmCYP78A5* regulates SS and weight (Du et al., 2017b), and the expression of *GmPSKγ1* increases SS and yield (Yu et al., 2019). *GmSWEET10a (Glyma.15G049200)* is related to SS increase and has a positive relation with the oil content but a negative relation with the protein content of the seeds (Wang et al., 2020). Genes located in the ST1 locus, mainly *Glyma.08g109100*, affect the seed thickness and increase the oil content of the seeds through the pectin biosynthesis pathway (Li et al., 2022a). *GmSSS1*, located on chromosome 19, is related to increased SS (Zhu et al., 2022), and *GmGA3ox1*, encoding the gibberellin synthesis pathway enhanced the photosynthesis, resulting in increased SW (Huang et al., 2022). Similarly, Lu et al. (2017) reported that *GmBZR1* in transgenic soybean, associated with the *PP2C-1* allele, increases SW and SS by facilitating the accumulation of dephosphorylated *GmBZR1*(Lu et al., 2017). Recently, a novel SS gene, *Glyma.08G309000*—named *Novel Seed Size* (*NSS*)—was found to control seed development in soybean (Zhang et al., 2023). The major genes related to the soybean seed yield and associated traits are listed in **Table 3**.

**Table 3:** List of reported genes functionally characterized for soybean yield and related traits.

Gene	Method	Gene ID number	Functional annotation	Associated trait	Reference
*D1*	CT/Transgenic	*Glyma.01g214600*	Stay-green gene 2 (SGR2)	Yield (chlorophyll degradation)	(Fang et al., 2014; Li et al., 2016; Slattery et al., 2016)
*PD1*	RNAi/CRISPR/Cas9	*Glyma.01g240100*	HD-Zip transcription factor	Increase stress resistance, yield	(Liu et al., 2020c)
*GmCYP78A57*	KO	*Glyma.02g119600*	Cytochrome P450 CYP2 subfamily/Homolog of AtCYP78A6/KLU	Yield (seed size)	(Zhao et al., 2016)
*GmPSKγ1*	OX	*Glyma.02g126200*	Phytosulfokine-gamma1	Yield seed size and weight	(Yu et al., 2019)
*GmFAD3*	RNAi LOF	*Glyma.03g056700 (a)* *Glyma.07g151300 (b)* *Glyma.11g1 74100 (c)*	Delta (12)-fatty acid dehydrogenase	Bigger seed and increased yield/increased jasmonic acid content	(Singh et al., 2010) (Zhang et al., 2008)
*GmCRY1a*	CRISPR/Cas9-mutagenesis	*Glyma.04g101500*	Cryptochrome 1a	Flowering/Yield/Domestication	(Lyu et al., 2021)
*GmCYP78A10*	_	*Glyma.05g019200*	Cytochrome P450 CYP2 subfamily/Flavonoid 3’,5’-hydroxylase	Yield (seed size)	(Wang et al., 2015b)
*GmCYP78A5*	OX	*Glyma.05g019200*	Cytochrome P450 CYP2 subfamily/Flavonoid 3’,5’-hydroxylase	Increased seed size and weight	(Du et al., 2017b; Du et al., 2017b)
*CTP*	LOF	*Glyma.05g022400*	DIACYLGLYCEROL KINASE 1	Yield/Flowering	(Zhao et al., 2017)
*SoyWRKY15a*	Transgenic	*Glyma.05g207100*	WRKY transcription factor	Yield (seed size)/Domestication	(Gu et al., 2017)
*GA20OX*	OX	*Glyma.07g081700*	Gibberellin 20 oxidase 2	Yield (seed size/weight)/Seed quality (oil content)/Domestication	(Lu et al., 2016)
*ST1*	CRISPR/Cas9-LOF	*Glyma.08g109100*	UDP-D-glucuronate 4-epimerase	Significantly decreased seed length, width, and thickness in soybean	(Li et al., 2022b)
*GmSWEET10b*	OX/CRISPR/Cas9 mutations	*Glyma.08g183500*	Sugar efflux transporter SWEET24	Seed quality (oil and protein contents)	(Wang et al., 2020)
*GmWRI1b*	OX	*Glyma.08g244200*	Ethylene-responsive transcription factor WRI1b	Seed quality (oil content)/increase in the pod and seed number per plant	(Guo et al., 2020)
*NSS*	LOF	*Glyma.08g309000*	Unknown protein/Peptidase-c1 domain/DNA helicase RuvA subunit	Reduced seed size, lower 100-seed weight, and reduced area of integument cells	(Zhang et al., 2023)
*GmSDP1-2*	Transgenic	*Glyma.10g105200*	Triacylglycerol lipase SDP1	Seed quality (oil content)/increased seed weight	(Kanai et al., 2019)
*GmBS1*	RNAi	*Glyma.10g244400*	Group II member of the TIFY family of transcription regulators	Yield (seed size)	(Ge et al., 2016)
*HSFB2b*	OX	*Glyma.11g02800*	Class B heat shock factor	Seed quality (flavonoid biosynthesis)/larger and higher 100-seed weight	(Bian et al., 2020)
*D2*	CT/Transgenic	*Glyma.11g029800*	Stay-green gene 2 (SGR2)	Yield (chlorophyll degradation)	(Fang et al., 2014; Li et al., 2016; Slattery et al., 2016)
*PS*	RNAi/CRISPR/Cas9	*Glyma.12g187200*	Unknown protein	Yield/Stress resistance	(Liu et al., 2020c)
*GmPDAT*	OX/RNAi	*Glyma.13g108100*	Phospholipid: diacylglycerol acyltransferase	Overexpression increases seed size/RNAi reduces seed size and plant height	(Liu et al., 2020b)
*GmSWEET10a*	CRISPR/Cas9-mediated OX	*Glyma.15g049200*	Sugar efflux transporter SWEET39	Seed quality (oil and protein contents)	(Wang et al., 2020)
*GmWRI1a*		*Glyma.15g221600*	Ethylene-responsive transcription factor WRI1a	Seed quality (oil and fatty acid)/higher seed weight per plant and per 100 seeds	(Chen et al., 2018)
*GmCIF1*	RNAi	*Glyma.17g040400*	Cell-wall inhibitor of beta-fructosidase	Yield (seed weight/size)	(Tang et al., 2017)
*GmKIX8-1*	LOF	*Glyma.17g112800*	Unknown protein	Yield (seed size)	(Nguyen et al., 2021)
*PP2C-1*	OX	*Glyma.17g221100*	Putative phosphatase 2C protein	Yield (seed weight)/Domestication	(Lu et al., 2017)
*GmSOC1*	LOF	*Glyma.18g224500 (a) Glyma.09g266200 (b)*	MADS-box family, belonging to the TM3 MIKCc subfamily	Increased plant height, node number, and grain weight per plant	(Chen et al., 2020b)
*GmSDP1-3*	Transgenic	*Glyma.19g132900*	Triacylglycerol lipase SDP1	Seed quality (oil content)/increased seed weight	(Kanai et al., 2019)
*GmCYP78A72*	OX	*Glyma.19g240800*	Cytochrome P450 CYP2 subfamily/Homolog of AtCYP78A7/KLU	Yield (seed size) increase seed size	(Zhao et al., 2016)
*GmNAP1*	LOF	*Glyma.20g019300*	NCK-associated protein	Reduced seed size and plant height	(Campbell et al., 2016; Tang et al., 2020)
*GmLEC2a*	Transgenic	*Glyma.20G035800.1*	B3 domain transcription factor	Seed development and biosynthesis of seed storage substances	(Manan et al., 2017)
*Ln/GmJAG1*	LOF	*Glyma.20g116200*	C2HC zinc fingers transcription factor	Yield (seed number per pod)	(Jeong et al., 2011; Jeong et al., 2012; Fang et al., 2013)
*GmABI3*	Transgenic	*Glyma.08G357600.1*	B3 domain-containing transcription factor ABI3	Lipid biosynthesis and stress tolerance	(Manan and Zhao, 2020)
*GmOLEO1*		*Glyma.20g196600*	Oleosin-encoding gene	Seed quality (oil content)	(Zhang et al., 2019)
*CRY2a*	RNAi	*Glyma.20g209900*	Cryptochrome 2c	Yield (leaf senescence)	(Meng et al., 2013)
*GmGA3ox1*	Transgenic	*Glyma.07g033800*	Gibberellin 3β-hydroxylase	Yield (seed size/seed weight)	(Hu et al., 2022)

OX, overexpression; LOF, loss of function, Gene ID from Glycine max Wm82.a2.v1.

## 4 Molecular networks regulating seed size

**Figure 3:**
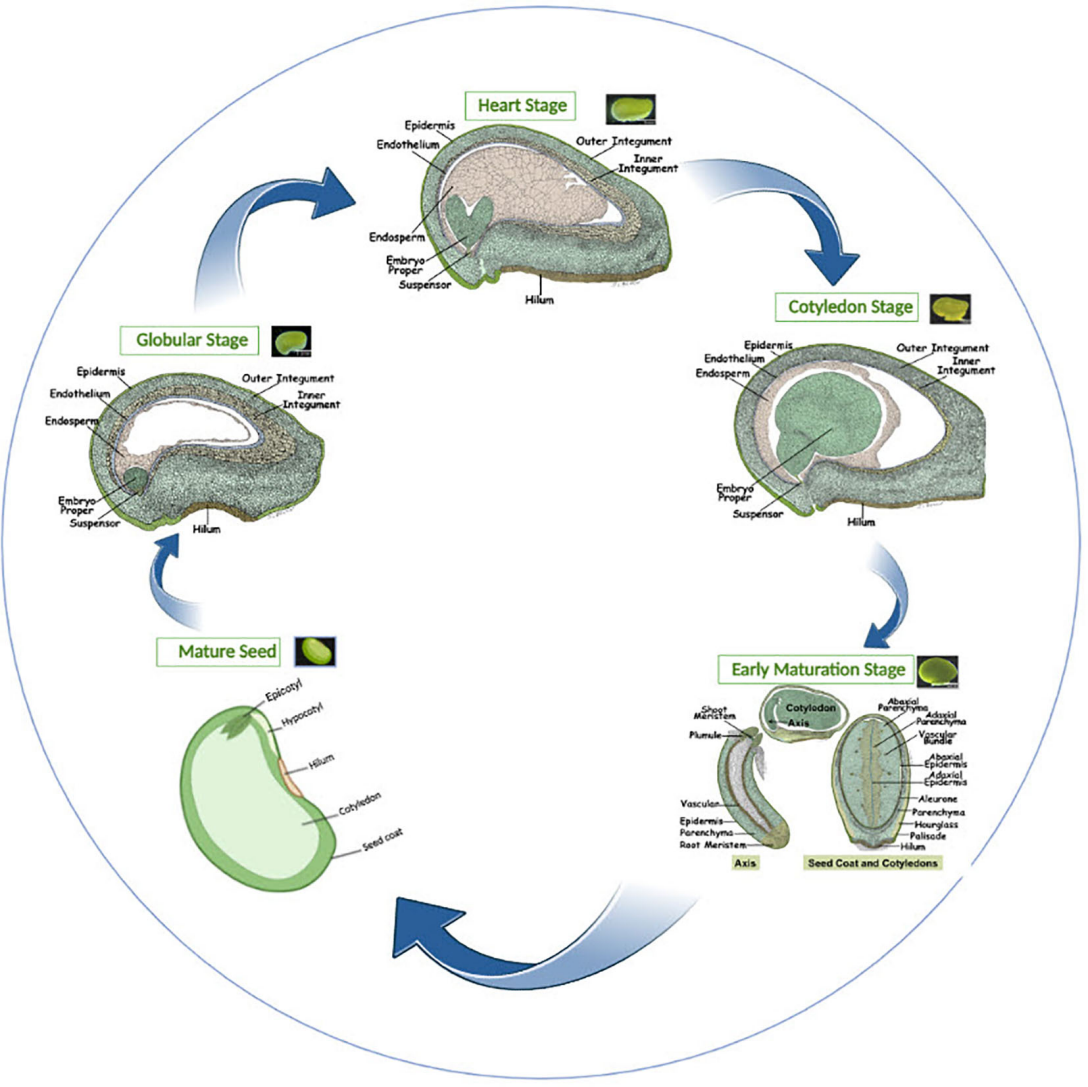
Illustrations of different stages and processes of soybean seed development from the globular to the mature seed stage. Globular stage to early maturation stage images adapted from http://seedgenenetwork.net/soybean (Images of globular to early maturation stage were hand-drawn by Sharon Lee Belkin (Morphographics).

Soybean SS is an important trait in crop yield. Increasing soybean yield per unit area is a primary breeding objective and a challenging task for the scientific community. Soybean SS, SW, and its protein and oil content are always been a priority of plant breeders to improve where significant improvements have been noted throughout time, and still going on, resulting in better seeds; phenotypically and nutritionally. Typically, plant seeds are composed of three essential parts; seed coat, diploid embryo, and triploid endosperm, which regulate seed development and subsequently determine SS (Garcia et al., 2005; Haughn and Chaudhury, 2005). The endosperm and embryo of dicots like *Arabidopsis* and soybean proliferate during seed development, and this pattern continues until the seed matures and desiccates (**Figure 3**). The maternal ovule simultaneously experiences controlled development to provide room for the developing embryo and endosperm, and its integuments eventually become the mature seed’s coat at a later developmental stage (**Figure 3**). The seed size directly correlates with the endosperm’s cell division (Garcia et al., 2005). Plant signaling mechanisms that control SS include the ubiquitin-protease route, mitogen-activated protein kinase (MAPK) signaling, transcriptional control, sugar, G protein signaling, HAIKU (IKU) pathways, and plant hormone signaling (Ruan et al., 2012; Li et al., 2019b; Pandey, 2019). Based on previous studies, we have briefly illustrated the major regulatory networks involved during seed development, ultimately determining the soybean SS.

**Figure 4:**
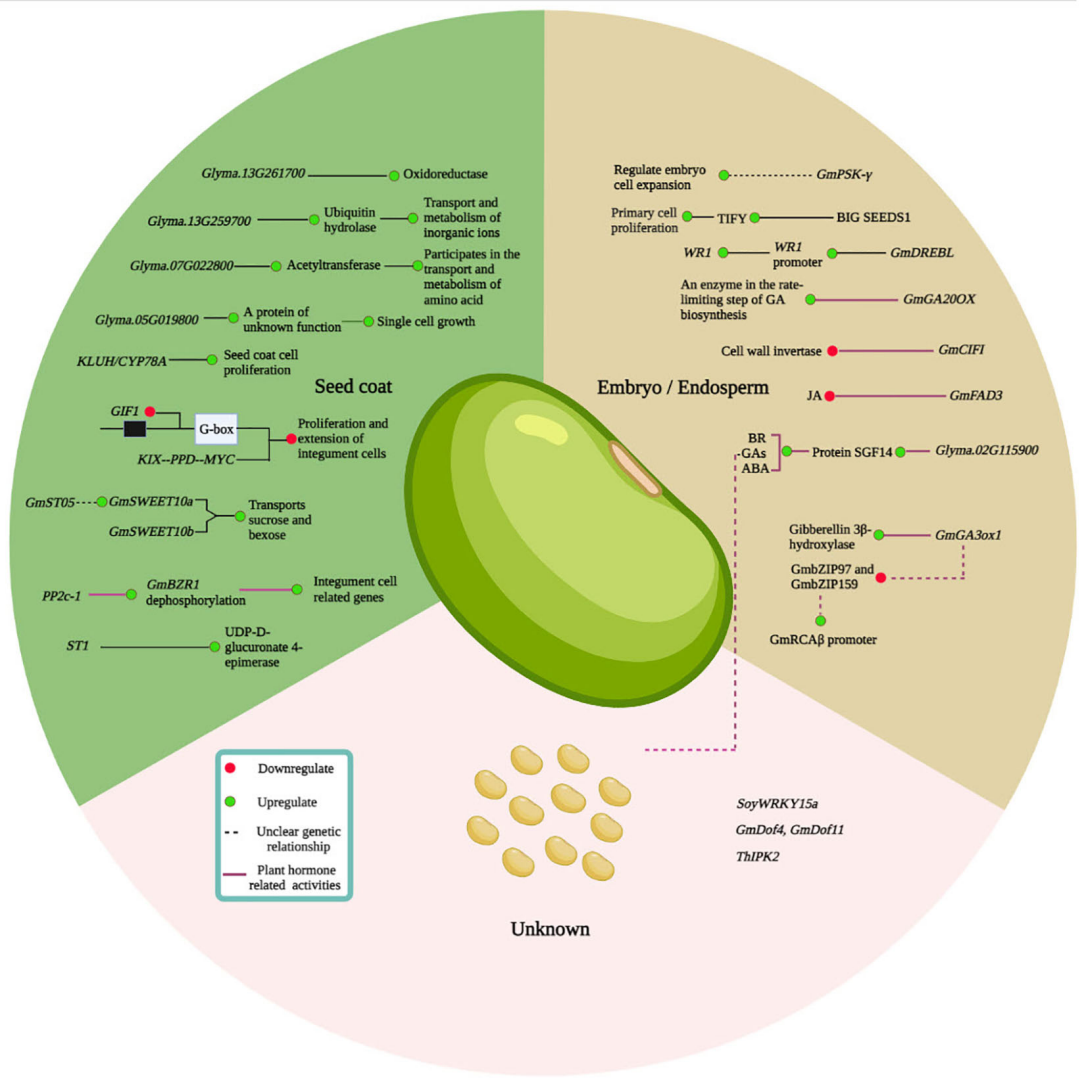
Overview of a regulatory network that determines soybean seed size (SS). The soybean SS determination mainly involves the ubiquitin-protease factors, mitogen-activated protein kinase (MAPK) signaling, G protein, transcriptional, carbohydrate, or sugar regulation, HAIKU (IKU) pathways, and plant hormone regulation. Based on the literature, key genes involved in SS regulation are explained in section 3. The green lines represent the activities in seed coat development; the black lines indicate the development of embryo/endosperm; the red represents the hormonal regulations; and the gray line demonstrates the regulation of an unknown location.

Besides its ultimate importance, the molecular processes underpinning soybean SS, shape, and weight are poorly understood. Nevertheless, several soybean genes have been identified and functionally characterized to be involved in regulating SS and SW (**Table 3**). Among them, some genes are identified for embryo and endosperm development, seed coat, and plant hormones, and others are related to different seed developmental stages, all of which are essential in the regulation of soybean SS (**Figure 4**).

### 4.1 Genes regulating embryo or endosperm

Although the exact mechanism and pathways regulating seed size are poorly studied and widely unexplored, though some transcriptional regulators that can control SS by regulating embryo development have been reported. For example, *BIG SEEDS1* (*BS1*)belongs to a group II member of the TIFY TF family and encodes the plant-specific transcriptional regulator TIFY, which plays a vital role in controlling the size of seeds, pods, and leaves via a regulatory module that targets cell proliferation (Ge et al., 2016). Ge et al. (2016) reported increased SS when the soybean *BS1* homolog is suppressed or downregulated. The study also indicated that in *Medicago truncatula big seeds1-1* (*mtbs1-1*) mutants, the expression of *GIF1*, *GROWTH REGULATING FACTOR 5* (*GRF5*), and core cell cycle genes were upregulated, and the embryo size was significantly increased. These results indicate that *BS1* is a negative regulator related to embryonic cell proliferation. Similarly, the IKU pathway mainly regulates early endosperm development and includes the IKU1, IKU2, MINISEED3 (MINI3), and SHORT HYPOCOTYL UNDER BLUE1 (SHB1) genes which reduced endosperm growth and small seeds. As IKU1 encodes a protein containing the plant-specific VQ motif and is expressed in early endosperm and in the central cell, which thereby it was consider an essential regulatory element for seed size regulation, where the IKU2 encoded a leucine-rich repeat (LRR) kinase, and its overexpression led to an increase in seed size, weight, and oil content (Fatihi et al., 2013). Additionally, loss of function of OsNAC129 was found to significantly increase grain length, weight, apparent amylose content (AAC), and plant height, while overexpression of OsNAC129 had the opposite effect. The expression of OsNAC129 was linked to induced by ABA, and overexpression of OsNAC129 in plants reduced sensitivity to exogenous BR, thereby indicating that overexpression of OsNAC129 negatively regulates seed development and plant growth and participates in the BR signaling pathway (Jin et al., 2022). In addition, studies demonstrated that the DREB-type transcription factor gene *GmDREBL* is essential for seed oil accumulation (Zhang et al., 2016b). *GmDREBL* is located in the nucleus and can activate transcription. It can bind to the promoter region of *WR1* to activate its expression. An overexpression study of *GmDREBL* in *Arabidopsis* indicated a specificity in stamens, embryos, and cotyledon organs and a significant increase in SS. The average expression of *DREBL* in cultivated soybean was higher than in wild soybean, suggesting that the trait might have been artificially selected to increase yield and oil content during soybean domestication (Zhang et al., 2016b). In addition to the myeloblastosis (MYB) family in this context, recent reviews and references describe the transcriptional factors regulating SS in *Arabidopsis* and other crops (Dwivedi et al., 2021; Alam et al., 2022).

### 4.2 Genes related to plant hormones

Some genes affect seed development by regulating the synthesis and metabolism of plant hormones. For example, the *Arabidopsis AtGA20OX* and its soybean homolog *GmGA20OX* encode the gibberellin oxidase GA-20. *GmGA20OX*, located in the soybean SW locus, was significantly correlated with HSW (Lu et al., 2016). As a multifunctional enzyme, *GA20OX* regulates gibberellin (GA) synthesis and metabolism, which are crucial in reproductive and physiological processes (Lu et al., 2016). Similarly, (Shi et al., 2020) Shi et al. (2020), successfully cloned GW6 (GRAIN WIDTH 6), a QTL affecting grain size, which positively influenced grain size and weight, and was encoded as GA-induced GAST family protein. Additionally, the GA content of young panicles was decreased by GW6 knockout. GW6’s transcript abundance, grain breadth, and grain weight are all influenced by a natural variation in the CAAT-box of the promoter. Transgenic plants overexpressing *Arabidopsis* GA2-oxidase gene (AtGA2ox8) in Brassica napus L. were shown to exhibit a significant increase in seed yield by 9.6–12.4% (Zhou et al., 2012). Gibberellic acid-stimulated *Arabidopsis* 4 (GASA4) is one of the 14 members of the small polypeptide family in *Arabidopsis*, regulating flowering and seed development and affecting seed size, weight, and yield. Furthermore, auxin is a crucial traditional phytohormone that plays crucial roles in a variety of processes related to plant growth and development, including grain size. Indole-3-acetic acid (IAA)-glucose hydrolase is encoded by qTGW6, which is a major QTL regulating rice grain weight. This enzyme produces free IAA. According to (Ishimaru et al., 2013), a loss-of-function TGW6 allele improved grain length and weight. TGW6 may be crucial in controlling pollen development, according to recent research that found it to be primarily expressed in pre-emergent inflorescences (Akabane et al., 2021; Kabir and Nonhebel, 2021). Auxin transport is regulated by a protein that is encoded by the auxin major response gene BG1. By encouraging cell division and elongation, it influences grain size (Liu et al., 2015) (Liu et al., 2015a). According to Hu et al. (2018) qTGW3/GL3.3, which codes for the SHAGGY-like kinase 41 (OsSK41), is a significant QTL for grain weight. A transcription repressor in the auxin pathway called OsARF4 is directly interacted with by OsSK41 and phosphorylated by it. ABA regulates various aspects of plant growth and development as well as responses to abiotic stress (Shi et al., 2021). LOS5/ABA3 is involved in ABA biosynthesis by encoding molybdenum co-factor sulfurase, which is required by aldehyde oxidase (AO) in the last step of ABA biosynthesis in plants. Transgenic plants overexpressing LOS5/ABA3 have been reported to show at least a 21% increase in seed yield compared to the wild type (WT) under drought stress conditions (Li et al., 2013b). OsAO3 is essential for the regulation of grain yield in rice, since osao3 mutant increases grain yield, while overexpression of OsAO3 reduces grain yield by affecting panicle number per plant, spikelet number per panicle, and spikelet fertility (Shi et al., 2021). Cytosolic ABA receptors PYRABACTIN RESISTANCE 1 IKE/REGULATORY COMPONENTS OF ABA RECEPTORS (PYL/RCARs) can regulate ABA-dependent gene expression in rice (Kim et al., 2014). Manan and Zhao (2020) reported that *Glycine max ABSCISIC ACID INSENSETIVE 3 (GmABI3*) is involved in lipid biosynthesis. The expression of *GmABI3* under environmental stress (heat and cold) and hormal stress (ABA and methyl jasmonate) was also studied. This research also identified a 34.9% increase in triacylglycerol (TAG) levels in transgenic *GmABI3/wildtype* seeds when compared to regular wildtype seeds. Furthermore, it revealed that this specific gene was accountable for producing long-chain fatty acids and generating TAG in a seed-specific manner. Constitutive expression of OsPYL/RCAR5 slightly reduces plant height and severely decreases seed yield under paddy field conditions, although abiotic stress tolerance is improved. Similarly, soybean *Glyma.02G115900* regulates multiple action pathways of plant hormones by affecting 14-3-3. Such pathways are complex and need further exploration and research. Recently, *GmGA30x1*, which encodes a gibberellin 3β-hydroxylase, displayed a positive SW and length regulation. The mutant *gmga30x1* displayed a reduced bioactive GA production and an enhanced net photosynthesis rate and Rubisco activity, leading to a reduced SW and an increased seed yield—through an increased seed number (Hu et al., 2022). The polyhydroxylated steroidal hormones known as BRs, which are specific to plants, regulate a variety of growth and developmental processes, including grain size (Li et al., 2018). As GSK2, which is a key negative regulator of the BR pathway, and a homolog of BIN2 in the GSK3/SHAGGY kinases family. As a result, increasing grain size and leaf angles were achieved by decreasing GSK2 expression (Tong et al., 2012). The main QTL for grain width, GW5, is composed of three haplotypes (Zhou et al., 2017a). The transcription factors OsBZR1 and DLT are released into the active state by GW5, which participates in the BR pathway by inhibiting GSK2 kinase activity and regulates grain width and weight (Liu et al., 2017a). Similarly, (Tang et al., 2017) reported that two invertase inhibitors, GmCIF1 and GmC/VIF2, had inhibitory activity *in vitro* through heterologous expression. Transcript analysis revealed that they were predominantly expressed in developing seeds and the ABA response. The silencing of *GmCIF1* significantly increased the CWI activity of mature seeds through processes that fine-tuned sucrose metabolism and pool strength and increased SW, hexose, starch, and protein accumulation (Tang et al., 2017), indicating a role for *GmCIF1* in the negative regulation of soybean SW. Other genes in soybean can affect SS by regulating other metabolites. For example, *GmFAD3*, an omega-3 fatty acid desaturase (FAD3), is involved in the biosynthesis of fatty acid and jasmonic acid (JA). A study indicated that *gmfad3*-RNAi silenced plants accumulate higher levels of JA, thereby increasing the susceptibility of soybean to bean pod mottle virus and producing larger and heavier seeds (Singh et al., 2010)

### 4.3 Other genes

Cultivated soybeans were successively domesticated from wild soybeans and generally displayed larger SS. Understanding the differences between cultivated and wild soybean traits, especially at the genomic level, can lead to genetic and cultivation improvement. In addition to the abovementioned regulatory pathways and genes, other genes are involved in regulating SS in soybean. For example, the expression level of *SoyWRKY15a* in wild soybean populations was positively correlated with SS (Gu et al., 2017). The coding sequences of *GmWRKY15a* (cultivated) and *GsWRKY15a* (wild) are identical, but the number of CT repeats in the 5’ untranslated region (5’UTR) is different, causing haplotype and, ultimately, SS variation (Gu et al., 2017). Previously, the regulatory role of WRKY transcription factors TRANSPARENT TESTA GLABRA 2 (TTG2) and MINISEED 3 (MINI3) in SS was demonstrated in *Arabidopsis* (Garcia et al., 2005; Li et al., 2013a). The loss of *TTG2* function in *ttg2* mutant plants causes impaired elongation of epidermal cells, impacting the endosperm and seed development (Garcia et al., 2005). Nonetheless, MINI3 can bind to the cytokinin oxidase 2 (CKX2) promoter, activate CKX2 expression, and control endosperm growth (Li et al., 2013a). However, further research is needed to fully understand the role of *SoyWRKY15* in SS and development.

A few transgenic studies have also provided evidence about genes influencing the SS. For example, Wang et al. (2007) studied soybean Dof-type transcription factor genes and found that transgenic *Arabidopsis* plants expressing *GmDof4* and *GmDof11* had slightly larger SS and SW and increased levels of lipid content compared to Col-0 (wild-type) plants, indicating that *GmDof4* and *GmDof11* affect soybean SW (Wang et al., 2007), and have a potential application value. Liu et al. (2012) introduced an inositol polyphosphate kinase gene from halophilic bacteria *(ThIPK2*) to soybean through Agrobacterium-mediated transformation, and the transformed soybean plants significantly improved their SS and stress resistance.

In summary, soybean seed size is regulated by multiple pathways and involves several molecular networks during seed development. An in-depth research is required to identify molecular mechanisms of SS development. However, based on recent advances at the molecular level, we illustrated a regulatory network that plays a role in the development of soybean SS. This information may be helpful in describing soybean SS and may open new opportunities for developing new soybean varieties with higher yield potential.

## 5 Challenges and perspectives

Soybean is one of the most important crops globally, and its yield has increased over the years but has become stagnant in the past few years. Challenges remain to be addressed to sustainably improve soybean yield and related traits. Moreover, the molecular mechanisms driving the fundamental biological processes involved in yield traits remain mostly unknown. Several studies using bi-parental mapping and association mapping identified the number of QTLs and QTNs for the soybean seed yield and related traits, and some of it has already been integrated into the SoyBase. However, a significant approach is lacking to successfully utilize identified QTLs by marker-assisted selection or breeding, because of the quantitative nature of the trait where a huge number of minor QTLs contribute to it which is difficult comparatively to the traits where one or few putative genes are involved, increasing predicament in molecular breeding. Previous studies comparatively used the low-density marker (SSR), confining low resolution and higher confidence interval for discovered SS/SW; other seed yield traits QTLs have not mined the causing candidate genes (Panthee et al., 2005; Gai et al., 2007; Kato et al., 2014; Kulkarni et al., 2016). Considering the complexity of traits and low inheritance of associated traits, it is challenging to detect stable QTLs. Moreover, few studies reported stable QTLs across the tested environment for soybean seed yield traits (Cui et al., 2020; Hina et al., 2020; Kumawat and Xu, 2021). Furthermore, validation of these identified and stable or previously reported QTLs in different genetic backgrounds is challenging, and very limited efforts have been made toward it. Several stable yield QTLs and genes have been identified and cloned in other crop species, such as rice (Xing and Zhang, 2010; He et al., 2022). However, progress toward functional characterization of seed yield-related QTLs or genes is lagging in soybean. Functional characterization of QTLs or genes reported for yield traits to develop high-yielding crops will be simpler if we can comprehend the genetic basis of each attribute and the regulatory network of seed yield traits (Tian et al., 2021; Zhang et al., 2022). Thus, perspectives to improve soybean seed yield should include the following steps. (1) In the soybean breeding program, effort must be given for the precise detection of phenotypic variation, selection or creation of genetically diverse resources, identification of stable QTLs or QTNs, and validation into diverse backgrounds. (2) Researchers should focus on understanding and identifying the genetics of soybean yield and related component traits using the high density and high-throughput markers (SNP) to further discover the candidate genes for traits. (3) These markers could then be used to develop molecular breeding tools that enable breeders to select plants and even perform the prediction breeding for desirable yield-related traits. (4) Studies should be conducted on soybean seed yield epigenetic changes, which may lead to finding ways to manipulate these changes to increase yield. Moreover, epigenetic changes can be influenced by environmental factors and profoundly impact plant growth and development. (5) In addition to genomics studies, transcriptomic and proteomic studies are needed for soybean seeds to identify genes and proteins that play key roles in seed development and yield. This information can be used to develop targeted breeding strategies or to engineer soybean plants to express specific genes or proteins that enhance seed yield. (6) Efforts need to be put in for the functional characterization of more genes associated with seed yield-related traits. (7) In addition to QTLs or genes responsible for key yield traits (SS, SW, and HSW), other traits like plant architecture (which is not discussed in this review), plant height, number of branching, number of internodes, NPP, number of seeds per pod, petiole angle, petiole length, and leaf size must be isolated and characterized using functional genomic investigations. The use of CRISPR/Cas9 technology for gene characterization for mentioned traits, where help can be taken from specific databases like SoyBase, could be a way forward to speed up the process. The CRISPR/Cas9 method has become a powerful tool with numerous uses in the areas of reverse genetics and crop enhancement. This pioneering technique has the potential to greatly improve the economic value and sustainability of crops in the face of biotic and abiotic stresses by precisely targeting specific genetic characteristics. At present, the CRISPR/Cas9 gene editing system has been successfully used in around 20 crop species, allowing for the enhancement of various desirable traits such as yield and resistance to biotic and abiotic stresses. In the last five years, major progress in genome editing research has mainly been made on important crops such as rice, wheat, maize, and soybean (Chaudhary et al., 2022) where focusing on the important yield related traits such as pod number, number of seeds, SS, SW, can bring enormous improvements in the crop yield. (8) Timely selection, introgression of superior yield traits, and pyramiding of high-yielding genes and other plant attributes may aid in developing a soybean cultivar with a significant yield increase. Besides genetic and genomic approaches, to combat the effects of climate change, disease, and pests on yield, new approaches are required, such as creating climate- and disease-resistant soybean cultivars, enhancing soil health, and implementing artificial intelligence for sustainable agriculture methods. Future soybean breeding efforts should prioritize the development of high-yielding varieties with enhanced agronomic traits to maximize soybean productivity. Breeding programs should aim to improve traits such as increased seed yield per plant, optimized seed size and weight, enhanced branching and podding characteristics, and improved stress tolerance. These advancements would contribute to the overall goal of achieving higher soybean yields and ensuring economic viability for soybean growers. Additionally, it is essential to emphasize the development of soybean varieties with improved resistance to biotic and abiotic stresses, including pests, diseases, drought, and temperature fluctuations. By incorporating traits associated with resistance and stress tolerance into breeding programs, breeders can mitigate yield losses caused by these challenges and improve overall crop performance. Furthermore, future breeding efforts should focus on harnessing the potential of emerging technologies, such as CRISPR/cas9 and marker-assisted breeding, to accelerate the breeding process and enhance precision in selecting desirable yield-related traits. This integration of cutting-edge tools and techniques can expedite the development of high-yielding soybean varieties with superior agronomic performance. By prioritizing the breeding objectives related to increasing soybean yield and yield-related traits, breeders can contribute to meeting the growing global demand for soybean products, ensuring food security, and promoting the economic prosperity of soybean production.

## Author contributions

RT, MI, wrote the manuscript. WK, AG, and RS prepared illustrations, figures, tables, and references. YK contributed critical comments to the draft. RT and YK conceptualized. YK critically edited, and approved the manuscript. All authors contributed to the article and approved the submitted version.
